# Psychosocial factors influencing dietary management in patients with type 2 diabetes and healthy adults: an ecological momentary assessment approach

**DOI:** 10.3389/fpsyg.2024.1464542

**Published:** 2025-01-07

**Authors:** Junichi Saito, Hiroaki Kumano

**Affiliations:** ^1^Comprehensive Research Organization, Waseda University, Tokyo, Japan; ^2^Graduate School of Human Sciences, Waseda University, Saitama, Japan

**Keywords:** type 2 diabetes, ecological momentary assessment, flash glucose monitoring system, dietary management, physical activity, psychosocial factors

## Abstract

**Background:**

Dietary management in diabetic patients is affected by psychosocial factors and the social-environmental context. Ecological momentary assessment (EMA) allows patients to consistently report their experiences in real-time over a certain period and across different contexts. Despite the importance of dietary management, only a few EMA studies have been conducted on dietary management and psychosocial factors in patients with type 2 diabetes; further evidence must be gathered. Therefore, this study examined dietary management and psychosocial factors using EMA, comparing type 2 diabetes patients with healthy adults.

**Methods:**

A total of 20 patients with type 2 diabetes and 16 healthy adults underwent EMA. Relying on event-contingent recordings, this study evaluated the participants’ mood (e.g., anxiety, anger, vigor), appetite (hunger, craving), meal types (e.g., breakfast), location (e.g., eating out), companions (e.g., family), and dietary lapses (e.g., I ate a larger portion of a meal or snack than I intended) before and after meals. Dietary lapse recording after meals was paired with psychosocial data before meals. Only the type 2 diabetes patients used a sensor-based glucose monitoring system (Freestyle Libre Pro, Abbot) and wearable activity monitors (GT3X-BT, ActiGraph).

**Results:**

The EMA produced a total of 4,254 responses. Dietary lapse predicted two-hour postprandial glucose through a sensor-based glucose monitoring system. Multilevel logistic regression analyses were performed. For diabetes patients, dietary lapse was affected by vigor, fatigue, and cravings before eating. Meanwhile, for healthy adults, only fatigue before meals affected dietary lapse, and increased vigor from dietary intake was associated with dietary lapse. In both type 2 diabetes patients and healthy adults, eating-out situations were linked to dietary lapse.

**Conclusion:**

The results suggest differences in psychosocial factors influencing dietary lapse between patients with type 2 diabetes and healthy adults. EMA is well suited to assess psychosocial factors that drive dietary management in diabetic patients. This study further discussed the possibility of individual approaches using EMA data.

## 1 Introduction

Type 2 diabetes, which accounts for 90% of all diagnosed cases, is a long-term illness with severe individual and societal effects. Those living with type 2 diabetes must perform diabetes self-care (van den Arend et al., 2000), a critical component of which is dietary management. This includes proper nutrition therapy and individualized meal planning, which can improve glycemic control and prevent complications (Powers et al., 2015; Evert et al., 2019). Effective diabetes self-management can help individuals maintain their blood glucose levels within a target range, which is essential for preventing or delaying diabetes-associated complications (Deakin et al., 2006). However, many type 2 diabetes patients encounter difficulties in self-management for different reasons, such as a lack of knowledge or education (Ernawati et al., 2021) and psychological factors (Whittemore et al., 2005). In Japan, experts recommend HbA1c levels below 7% to prevent diabetic complications (Araki et al., 2020), but approximately half of diabetic patients in the country exceed those levels.

Considerable research has focused on discovering psychosocial factors that could be targets for interventions to improve diabetes self-care. Psychosocial factors include psychological and social aspects that shape an individual’s thoughts, feelings, and behaviors (Young-Hyman et al., 2016). For patients with type 2 diabetes, psychosocial factors include stress, depression, social support, self-efficacy, diabetes-related stigma, and cognitive impairment, which can affect their ability to effectively manage their condition (Gonzalez et al., 2015). Studies have also observed a link between diabetes self-management and stress (Gazmararian et al., 2009), depression (Jerant et al., 2005; Egede and Ellis, 2008), social support (Nam et al., 2011), and self-efficacy (Qiu et al., 2012). However, most of these studies are cross-sectional and use retrospective questionnaires; only a few have examined moment-to-moment associations between psychosocial factors and diabetes self-management. Diabetes self-management occurs in the context of individuals’ daily lives. Hence, it is necessary to capture and model within-person variability in diabetes self-management (Wooldridge et al., 2022). Ecological momentary assessment (EMA; Shiffman et al., 2008) may be a solution to this problem.

EMA allows patients to consistently report real-time experiences in real-life situations over a specific period and across different contexts. EMA sampling and assessment schemes can be roughly classified into signal- and event-contingent recordings (Shiffman et al., 2008). Signal-contingent recording is a method where participants respond to prompts or signals delivered at random or fixed intervals, which is especially suitable for measuring variables that fluctuate over time, such as mood and anxiety (Hall et al., 2021). Meanwhile, event-contingent recording is particularly suited for measuring infrequent or irregular events, such as dietary lapses and caloric intake (Battaglia et al., 2022), and allows for the detailed tracking of occurrences whose patterns are unpredictable, making it ideal for studying behaviors or experiences that vary significantly from person to person or within the same person over time.

Key features of EMA include the collection of data on an individual’s recent or current state through multiple evaluations over time. This approach allows for an examination of variability within an individual (i.e., within-person analysis), which accounts for more of the variance in diabetes self-management behaviors as opposed to between-person analyses. For instance, Mulvaney et al. (2018) found that within-person psychosocial factors were responsible for most of the variance in missed self-monitored blood glucose checks and insulin administration among adolescents with type 1 diabetes. This highlights the importance of EMA when considering within-person variability in the analysis of diabetes self-management behaviors.

A recent systematic review of EMA studies on psychosocial factors in diabetes extracted 10 studies that mostly involved white adolescent patients with type 1 diabetes (Nam et al., 2021). Time-varying psychosocial factors such as negative affect and stress were associated with missed insulin injections and poor adherence to glucose monitoring. Two studies examined type 2 diabetes. Miller et al. (2016) assessed the feasibility EMA in tracking the intake of low glycemic index foods. Only one of the studies, Inada et al. (2019), discussed diabetes self-management and psychosocial factors. Inada et al. (2019) showed a positive association between stress and caloric intake from snacking and a negative association between anxiety and depressed mood and caloric intake from regular meals. They also demonstrated that caloric intake was greater when eating with others than when eating alone as well as when eating out than when eating at home. However, this systematic review noted that the small number of dietary management studies makes it difficult to reach general conclusions.

Despite the significance of dietary management, EMA studies on dietary management and psychosocial factors in patients with type 2 diabetes have been minimal, which requires further evidence. Therefore, this study examined dietary management and psychosocial factors using EMA and compared type 2 diabetes patients with healthy adults, which may reveal diabetes-specific psychosocial factors that influence dietary management. A comprehensive understanding of patients’ dietary management might significantly enhance the effectiveness of diabetes control strategies.

## 2 Methods

### 2.1 Participants

The participants were healthy adults and outpatients with type 2 diabetes diagnosed according to Japan Diabetes Society criteria. The type 2 diabetes patients were aged 20–69, had been visiting the internal medicine clinic for at least 6 months, and consented to participate in this study. These participants were recruited through posters displayed in the clinic. Exclusion criteria were (a) inability to understand or read Japanese well; (b) with serious diabetic complications (retinopathy, nephropathy, neuropathy); (c) with severe psychiatric disorders requiring specialized care; (d) with Alzheimer’s disease and vascular or other types of dementia; and (e) other cases deemed inappropriate by the attending physician. Similarly, healthy adults aged 20–69 years, with no significant physical or psychiatric disorders, and interested in dietary management were included in this study.

### 2.2 Measures

#### 2.2.1 Self-report questionnaires

The Japanese version of the Patient Health Questionnaire (PHQ-9) is a nine-item self-report questionnaire for measuring depression symptoms (Muramatsu et al., 2007). Items are rated on a four-point Likert-type scale ranging from 0 to 3, and total scores can range from 0 to 27, with higher scores indicating greater depression. Total scores can then be interpreted as suggesting no depression (0–4), mild depression (5–9), moderate depression (10–14), moderately severe depression (15–19), or severe depression (20–27). A cut-off score of 10 indicates a possible depressive disorder diagnosis.

Meanwhile, the Japanese version of the Dutch Eating Behavior Questionnaire (DEBQ) is a 33-item self-report questionnaire designed to measure emotional eating, external eating, and dietary restraint (Imada, 1994). Items are rated on a five-point Likert-type scale, ranging from 1 (never) to 5 (very often). The dietary restraint subscale contains 10 items (e.g., “Do you take into account your weight with what you eat?”), the emotional eating subscale 13 items (e.g., “Do you have a desire to eat when you are disappointed?”), and the external eating subscale 10 items (e.g., “If you see others eating, do you also have the desire to eat?”).

#### 2.2.2 Ecological momentary assessment

A two-week EMA was conducted on type 2 diabetes patients and healthy adults who provided consent to participate in this study. First, we confirmed that they could receive e-mails via smartphone and understand the questions and had no difficulty answering them. We then created a response form using Google Forms and asked them to access the form from an e-mail containing its URL.

The EMA in this study consisted of signal- and event-contingent recordings. Signal-contingent recording involved random notifications at 30-min intervals around the following times: 10:30, 14:30, and 20:30. If the participants failed to respond within 30 min, the recording opportunity was canceled. Meanwhile, event-contingent recording involved asking the participants questions immediately before and after their meals and upon waking up.

The participants were asked about the following at all response times: (1) Mood: The participants took the Japanese version of the Profile of Mood States (Yokoyama et al., 1990), and their responses followed a seven-point scale from “1: not at all” to “7: very strongly” regarding their degree of “tension-anxiety,” “depression,” “anger-hostility,” “vigor,” “fatigue,” and “confusion,” with one item each. Values for “stress” were obtained by subtracting the value for “vigor” from the sum of those for “anxiety,” “depression,” “anger,” “fatigue,” and “confusion.” Furthermore, “daily stress” was calculated using the average “stress” score of the EMA, which consisted of signal-contingent data for that day. (2) Appetite: Participants’ “hunger” and “craving” were assessed using the EMA for appetite (Kikuchi et al., 2015), with scores ranging from “1: not at all” to “7: very strongly.”

In addition, the following items were asked only immediately before meals: (3) Meal types: multiple-choice questions were asked for “breakfast,” “lunch,” “dinner,” and “snacks.” (4) Occasions: multiple-choice questions were asked regarding “at home,” “at work,” “eating out,” and “others.” (5) Companions: multiple-choice answers were obtained regarding “alone,” “family,” “friends,” “colleagues,” and “others.”

Moreover, the following items were asked only immediately after meals: (6) Dietary lapse: Following Forman et al. (2017), the participants were asked to select from “I ate a larger portion of a meal or snack than I intended,” “I ate when I had not intended to eat,” “I ate a type of food that I intended to avoid,” and “none of the above events” in a multiple-choice format. “Dietary lapse” in this study was defined as the selection of any option other than “none of the above events,” indicating noncompliance with appropriate dietary management.

Furthermore, the following items were asked only immediately after waking up: (7) Sleeping duration: The participants were asked the questions “What time did you go to bed?” and “What time did you get up?” This study calculated sleep duration based on these questions.

#### 2.2.3 Flash glucose monitoring system

The FreeStyle Libre Pro^™^ flash glucose monitoring system (Abbott Diabetes Care, Alameda, CA) measures changes in glucose levels. Unlike traditional glucose monitoring systems requiring finger pricks, this system uses a small sensor applied to the back of the upper arm that reads glucose levels from the interstitial fluid beneath the skin in the 40–500 mg/dL range every 15 min for up to 14 days. Sensor data is downloaded into the reader device. Although the sensor is single use only, the reader can collect data from multiple sensors. A graphical summary is then generated by connecting the reader to a computer with the associated software. This summary includes average daily glucose levels, time spent within the target glucose range, time below the target glucose range, and time above the target glucose range per day. This study used the participants’ average daily glucose levels and manually calculated their two-hour postprandial glucose.

#### 2.2.4 Accelerometers

GT3X-BT accelerometers (Actigraph, Pensacola, FL, United States) measure physical activity. The participants wore these devices on their waist throughout their waking hours except during water activities such as bathing. The ActiLife 6 software, developed by ActiGraph, was used to initialize the accelerometers and analyze the collected data. An epoch duration of 60 s was selected, with inactivity periods defined as 60 or more minutes of continuous readings of zero. According to Freedson et al.’s (1998) established cut-off points, physical activity levels were classified per minute into sedentary (0–99 counts per minute [CPM]), light (100–1951 CPM), moderate (1,952–5,724 CPM), vigorous (5,725–9,498 CPM), and very vigorous (more than 9,499 CPM). In this study, physical activity assessment was based on daily moderate-to-vigorous physical activity (MVPA; 1,952 CPM or more).

### 2.3 Statistical analysis

Demographic data underwent *t*-tests or chi-square tests to compare type 2 diabetes patients with healthy adults.

Because EMA data had a nested structure in which each participant had repeated recordings, multilevel modeling was used for statistical analyses. This study focused on the intraindividual level and individual changes and variability over time as opposed to interindividual differences, which are variations between individuals. In multilevel analysis, multiple observations obtained from the same individual are related, resulting in autocorrelation between the observations. Ignoring this autocorrelation can lead to underestimation of standard errors and an increased probability of committing a Type I error. Therefore, random intercept and random slope models are used in multilevel analysis to model the within-person autocorrelation properly. This study used random intercepts in multilevel modeling. Because of sample size limitations, this study did not use random slopes.

Before conducting the multilevel analyses, we examined whether the participants’ characteristics influenced their dietary lapse. To this end, we performed a univariate logistic regression analysis with each independent variable and dietary lapse as the dependent variable. Nominal variables were converted to 0 and dummy variables 1.

To ensure the validity of dietary lapses in patients with type 2 diabetes, this study analyzed a univariate model with dietary lapses as the independent variable and two-hour postprandial glucose levels as the dependent variable. The incremental area under the curve for two-hour postprandial blood glucose was calculated following the trapezoidal rule, that is, subtracting the baseline glucose value from each time point before calculating the area (Pruessner et al., 2003; Floch et al., 1990). This mathematical method approximates the area under a curve by dividing it into trapezoidal shapes and summing their areas to obtain a total value.

To investigate factors affecting dietary lapse, each dietary lapse recording was paired with a preceding psychosocial factor recording and treated as the dependent variable. Psychosocial factors were separately modeled as the independent variable. Odds ratios were calculated for these analyses; values greater than 1 indicated an association between a higher value of the preceding recording of psychosocial factors and a higher probability of dietary lapse. To examine whether each factor was independently associated, we analyzed multivariate models in which factors showing significant associations were independent variables. We also analyzed a univariate model with the change in psychological factors before and after meals as the dependent variable and dietary lapse as the independent variable.

To examine factors that influence daily glucose levels and time in target (80-140mg/dl), we analyzed a multivariate model in which “daily stress,” “daily MVPA,” “dietary lapse on that day,” and “sleeping duration” were independent variables and “daily glucose level” was the dependent variable. Glucose levels fluctuate for different reasons, especially the influence of medication, which cannot be ignored. However, records showed that all diabetes patients complied with their medications. Therefore, this study did not need to consider the influence of medication.

Based on Shiffman et al. (2008), records of participants with compliance rates below 70% were excluded from the data analysis to ensure quality data. Significance level was set at 0.05. Analyses were performed using R version 3.4.3 and a solver-off version of HAD17_202 (Shimizu, 2016).

## 3 Results

### 3.1 Participants’ characteristics

Table 1 shows the participants’ characteristics. The participants with type 2 diabetes were middle-aged, tended to be obese but had no severe complications, and were receiving diet therapy and oral medication. However, some patients reported poor glycemic control and depressive symptoms. For demographic characteristics, patients with type 2 diabetes and healthy adults were different only in terms of Body Mass Index (BMI).

**Table 1:** Participants’ characteristics.

	Type 2 diabetes (*N* = 20)	Healthy adults (*N* = 16)	*t*/*χ*^2^	*p* value
Age in years, *M* (*SD*)	53.05 (9.59)	49.50 (8.20)	1.19	0.24
Gender: *n* (%) female	8 (40%)	11 (68%)	2.89	0.11
Marital status, *n* (%)			1.85	0.49
Single	2 (10%)	0 (0%)		
Married	18 (90%)	16 (100%)		
Body mass index in k/m^2^, *M* (*SD*)	28.47 (6.82)	20.51 (2.86)	4.32	0.00
Diabetes duration years, *M* (*SD*)	9.21 (8.75)	–		
HbA1c, *M* (*SD*)	7.06 (0.60)	–		
Insulin, *n* (%)	0 (0%)	–		
Oral medication, *n* (%)	17 (81%)	–		
Diabetes complications, *n* (%)	0 (0%)	–		
PHQ-9, *M* (*SD*)	4.73 (3.92)	3.00 (2.98)	1.64	0.11
DEBQ for inhibitory eating, *M* (*SD*)	2.54 (0.66)	2.54 (0.60)	0.04	0.97
DEBQ for impulsive eating, *M* (SD)	2.66 (0.53)	2.70 (0.38)	−0.36	0.72
DEBQ for emotional eating, *M* (SD)	2.63 (0.70)	2.71 (0.52)	−0.35	0.73

### 3.2 Recording profiles

EMA data included 4,254 responses. The signal-contingent recordings involved a total of 568 patients with type 2 diabetes (adherence 75.13%, min 16.67%, max 100.00%), and 612 healthy adults (adherence 91.07%, min 61.90%, max 100.00%). The responses right after waking up consisted of a total of 203 patients with type 2 diabetes (mean 11.94 ± 1.22, min 11, max 14) and 200 healthy adults (mean 12.50 ± 1.54, min 10, max 14). The responses immediately before meals consisted of a total of 676 patients with type 2 diabetes (mean 39.76 ± 13.86, min 10, max 56) and 649 healthy adults (mean 40.56 ± 11.16, min 27, max 66). The responses immediately after meals consisted of 716 patients with type 2 diabetes (mean 37.35 ± 12.33, min 13, max 54) and 630 healthy adults (mean 39.37 ± 10.71, min 22, max 64). The responses paired with the before and after meals consisted of 462 patients with type 2 diabetes and 533 healthy adults. Meanwhile, this study excluded two patients with type 2 diabetes whose response rates were below 70%, one patient with type 2 diabetes who withdrew because of poor physical condition, and one healthy adult.

### 3.3 Psychosocial factors affecting dietary lapse

The probability of “dietary lapse” was 126 of 462 dietary episodes (27.27%) in type 2 diabetes patients and 118 of 532 dietary episodes (22.18%) in healthy adults. A univariate logistic regression analysis was performed before the multilevel analyses, with each participant’s characteristics as the independent variable and dietary lapse as the dependent variable. The results showed no significant associations between age (estimate = –0.02, SE = 0.02, *z* = –0.88, *p* = 0.39), gender (estimate = 0.00, SE = 0.42, *z* = 0.00, *p* = 0.99), BMI (estimate = 0.17, SE = 0.03, *z* = 1.44, *p* = 0.16), PHQ-9 (estimate = 0.09, SE = 0.06, *z* = 1.55, *p* = 0.13), DEBQ restrained eating (estimate = 0.18, SE = 0.39, *z* = 0.46, *p* = 0.65), DEBQ emotional eating (estimate = 0.17, SE = 0.57, *z* = 0.31, *p* = 0.76), DEBQ external eating (estimate = 0.66, SE = 0.39, *z* = 1.69, *p* = 0.10), and dietary lapse. Therefore, the subsequent multilevel analyses did not include these variables as covariates. The validity of “dietary lapse” in patients with type 2 diabetes was examined and showed that it predicts two-hour postprandial glucose (estimate = 100.70, SE = 38.74, *z* = 2.75, *p* = 0.00).

A univariate model was examined by multilevel logistic regression analysis in patients with type 2 diabetes. The results indicated that, as psychological factors, higher levels of “vigor,” “fatigue,” and “craving” immediately before meals were associated with a higher probability of “dietary lapse.” In addition, as a social factor, “eating out” was associated with a higher probability of “dietary lapse” as well. Furthermore, we examined the multivariate models using the abovementioned factors as independent variables. The results showed that all these factors were independently associated.

Similarly, we analyzed univariate models for healthy adults. The results indicated that, as a psychological factor, “fatigue” immediately before meals affected “dietary lapse” and that higher levels of “fatigue” immediately before meals were associated with a higher probability of “dietary lapse.” As social factors, “dinner,” “eating out,” and “social situation” were linked to a higher probability of “dietary lapse.” Furthermore, we examined multivariate models using the abovementioned factors as independent variables. The results showed that “dinner” and “social situation” independently affected “dietary lapse.” Table 2 presents these results.

**Table 2:** Psychosocial factors that influence dietary lapses.

	Type 2 diabetes (*Obs.* = 459)						Healthy adults (*Obs*. = 533)					
	Estimate	SE	*z*	*p*	OR	95% CI	Estimate	SE	*z*	*p*	OR	95% CI
Univariate model												
Psychological factors												
Tension-anxiety	−0.14	0.13	−1.08	0.28	0.87	[0.68, 1.11]	0.08	0.11	0.71	0.48	1.08	[0.87, 1.35]
Depression	−0.22	0.15	−1.47	0.14	0.81	[0.60, 1.08]	0.07	0.12	0.60	0.55	1.08	[0.84, 1.37]
Anger-hostility	−0.17	0.12	−1.34	0.18	0.85	[0.66, 1.08]	0.19	0.11	1.67	0.09	1.20	[0.97, 1.50]
Vigor	0.46	0.12	3.74	0.00	1.58	[1.24, 2.02]	0.11	0.11	0.97	0.33	1.11	[0.90, 1.38]
Fatigue	0.49	0.11	4.57	0.00	1.63	[1.32, 2.01]	0.21	0.08	2.51	0.01	1.23	[1.05, 1.45]
Confusion	0.09	0.14	0.62	0.54	1.09	[0.83, 1.43]	0.11	0.14	0.84	0.40	1.12	[0.86, 1.46]
Hunger	−0.03	0.08	−0.34	0.73	0.97	[0.83, 1.14]	−0.14	0.07	−1.89	0.06	0.86	[0.75, 1.00]
Cravings	0.34	0.09	3.88	0.00	1.34	[1.18, 1.66]	0.05	0.08	0.64	0.52	1.05	[0.90, 1.24]
Social factors												
Dinner	0.18	0.17	1.06	0.29	1.20	[0.86, 1.66]	0.74	0.23	3.22	0.00	2.10	[1.34, 3.29]
Eating out	1.32	0.56	2.37	0.02	3.74	[1.26, 11.11]	0.72	0.35	2.09	0.04	2.06	[1.04, 4.06]
Social situation	−0.44	0.38	−1.17	0.24	0.64	[0.31, 1.35]	1.18	0.42	2.82	0.00	3.25	[1.43, 7.37]
Multivariate model												
Psychological factors												
Vigor	0.48	0.13	3.75	0.00	1.62	[1.26, 2.09]	–	–	–	–	–	–
Fatigue	0.52	0.12	4.46	0.00	1.68	[1.34, 2.11]	0.16	0.09	1.85	0.06	1.18	[0.99, 1.40]
Cravings	0.23	0.09	2.52	0.01	1.26	[1.05, 1.52]	–	–	–	–	–	–
Social factors												
Dinner	–	–	–	–	–	–	0.70	0.24	2.92	0.00	1.26	[1.26, 3.23]
Eating out	1.58	0.61	2.61	0.01	4.86	[1.48, 15.91]	0.33	0.39	0.83	0.41	1.38	[0.64, 2.98]
Social situation	–	–	–	–	–	–	1.13	0.47	2.40	0.02	3.10	[1.26, 3.30]

Obs., observations.

### 3.4 Changes in psychological factors before and after eating

A univariate model was examined using multilevel regression analysis in patients with type 2 diabetes. The results showed that “dietary lapse” decreased “vigor,” “fatigue,” and “craving.” Similarly, univariate model analysis for healthy adults revealed that “dietary lapse” increased “vigor.” Table 3 shows these results.

**Table 3:** Changes in psychological factors before and after eating.

	Type 2 diabetes (*Obs*. = 459) Healthy adults (*Obs*. = 533)							
	Estimate	SE	z	*p*	Estimate	SE	z	*p*
Univariate model								
Psychological factors								
Δ Tension-anxiety	0.03	0.89	0.38	0.71	−0.03	0.20	−0.14	0.88
Δ Depression	0.12	0.09	1.29	0.20	0.06	0.12	0.53	0.60
Δ Anger-hostility	0.10	0.06	1.79	0.08	−0.07	0.21	−0.33	0.74
Δ Vigor	−0.57	0.23	−2.49	0.01	0.35	0.12	2.86	0.00
Δ Fatigue	−0.51	0.19	−2.67	0.01	0.02	0.07	0.31	0.75
Δ Confusion	−0.07	0.09	−0.80	0.42	0.03	0.06	0.44	0.66
Δ Hunger	−0.32	0.21	−1.54	0.13	0.09	0.40	0.24	0.81
Δ Cravings	−0.79	0.30	−2.60	0.01	−0.26	0.27	−0.96	0.34

Obs., observations.

### 3.5 Factors affecting daily glucose levels and time in target range

Multivariate model analysis via multilevel regression in type 2 diabetes patients indicated that higher levels of “daily stress” and the presence of “dietary lapse on that day” were independently associated with higher “daily glucose levels.” Meanwhile, higher levels of “daily MVPA” and longer “sleeping duration” were independently associated with lower “daily glucose levels.”

In addition to the factors influencing daily glucose levels, the multivariate model analysis also examined the factors affecting time in target range (80-140mg/dl), time below target (80mg/dl), and time above target (140mg/dl). The results showed that higher levels of daily MVPA and longer sleeping duration were independently associated with higher time in target range. Meanwhile, the presence of dietary lapses on that day was independently associated with lower time in target range. Daily stress did not have a significant association with time in target. For time above target (140mg/dl), higher levels of daily stress and the presence of dietary lapses on that day were independently associated with higher time above target. Conversely, higher levels of daily MVPA and longer sleeping duration were independently associated with lower time above target. None of the factors showed significant associations with time below target (80mg/dl). Table 4 displays these results.

**Table 4:** Factors that influence daily glucose levels and time in target.

	Type 2 diabetes (*Obs*. = 145)			
Multivariate model	Estimate	SE	*t*	*p*
Daily glucose levels				
Daily stress	1.08	0.32	3.40	0.00
Daily MVPA	−0.09	0.04	−2.34	0.02
Dietary lapses on that day	5.72	2.34	2.44	0.02
Sleeping duration	−1.94	0.76	−2.55	0.01
Time in target (80-140mg/dl)				
Daily stress	-0.46	0.32	-1.41	0.16
Daily MVPA	0.12	0.04	3.30	0.00
Dietary lapses on that day	-6.19	1.50	-4.15	0.00
Sleeping duration	3.04	0.76	4.00	0.00
Time below target (80mg/dl)				
Daily stress	0.74	0.35	2.07	0.12
Daily MVPA	0.05	0.04	1.11	0.27
Dietary lapses on that day	0.02	0.93	0.03	0.98
Sleeping duration	−0.29	0.34	−0.83	0.41
Time above target (140mg/dl)				
Daily stress	0.74	0.35	2.07	0.04
Daily MVPA	−0.14	0.03	−5.48	0.00
Dietary lapses on that day	5.56	1.42	3.92	0.00
Sleeping duration	−3.05	0.66	−4.66	0.00

Obs., observations; MVPA, moderate-to-vigorous intensity physical activity.

## 4 Discussion

This study performed EMA to examine dietary management and psychosocial factors, comparing type 2 diabetes patients with healthy adults. For the demographic data, no significant differences other than BMI were observed between type 2 diabetes patients and healthy adults. The close link between type 2 diabetes and obesity is well-known. A study in the United Kingdom showed that approximately 60% of individuals with type 2 diabetes had BMIs of 30 or higher (Daousi et al., 2006). However, compared to Western populations, the Japanese tend to develop insulin resistance at relatively lower levels of obesity, and there is a prevalence of lean type 2 diabetes (Sakurai et al., 2012). In this study, only a limited number of participants had BMIs of 30 or above.

Regarding EMA adherence, the signal-contingent recordings were lower in type 2 diabetes patients than in healthy adults. A systematic review found an 81.4% EMA adherence among studies focusing on key health behaviors (e.g., physical activity and dietary behavior) (Perski et al., 2022). In this study, adherence among type 2 diabetes patients was somewhat lower than in other studies but was still considered acceptable. In event-contingent recordings, no significant difference was observed in the number of recordings between type 2 diabetes patients and healthy adults. Dietary lapses in type 2 diabetes patients predicted their postprandial two-hour blood glucose integral. Factors influencing the postprandial two-hour blood glucose integral include “how much,” “what to eat,” and the order in which carbohydrates are consumed during a meal (Shukla et al., 2017). Although the order factor could not be examined in this study, the definition of dietary lapses is deemed to have maintained some level of validity.

Regarding psychosocial factors affecting dietary lapse, “vigor,” “fatigue,” and “craving” predicted “dietary lapse” in patients with type 2 diabetes. Meanwhile, only “fatigue” predicted “dietary lapse” in healthy adults. While many studies have investigated the relation between eating behavior and emotions, most have focused on negative emotions. Recently, however, studies have highlighted the importance of focusing on positive emotions (Bongers et al., 2013). For example, Boh et al. (2016) used different mood ratings in overweight and healthy-weight individuals before consuming high-calorie food, before consuming healthy food, and in nonmeal situations. The results showed that both overweight and healthy-weight participants’ positive emotions increased before consuming high-calorie food, with no association between negative emotions and food consumption. In the sole EMA study on dietary management among patients with type 2 diabetes, Inada et al. (2019) reported a positive correlation between stress and calorie intake from snacking and a negative correlation between anxiety and depressed mood and calorie intake from regular meals. In this study, fatigue, an element of stress, was also linked to dietary lapse. Also, although significant associations were not observed in this study, the consistent point was that anxiety and depression do not lead to inappropriate eating behaviors. Nevertheless, with regard to dietary management measures, studies have focused on different aspects—caloric intake and dietary lapses—which present challenges in direct comparisons of results. The current study also yielded unique findings, such as the role of positive emotions. Future research may need to examine positive emotions beyond the “vigor” in patients with type 2 diabetes in this study.

An association concerning “fatigue” was observed in both type 2 diabetes patients and healthy adults. Zhu et al. (2018) suggested that in managing the diets of patients with type 2 diabetes, fatigue may be an important factor. In Japan, Ohhinata and Nakamura (2009), who examined the connection between mood and food consumption in middle-aged diabetes patients, showed no association between depression or anxiety and food consumption but a strong positive link between fatigue and food consumption. Furthermore, using EMA, Inada et al. (2019) showed that depression and anxiety decrease food intake in patients with type 2 diabetes. In conclusion, our findings—derived from EMA and the literature—underscore the importance of investigating fatigue in the dietary management of individuals with type 2 diabetes. The association between fatigue and dietary lapses in both groups emphasizes the universal influence of the former on the latter. This highlights the need for interventions and strategies to manage fatigue and promote healthy eating habits not only for those with chronic conditions such as type 2 diabetes but also for the general population.

Kikuchi et al. (2015), measured “hunger” and “craving” and showed that in patients with type 2 diabetes, only “craving” predicts “dietary lapse.” Food craving refers to an intense and specific desire to eat a particular type of food (Weingarten and Elston, 1990) and is much stronger than normal hunger. It not only pertains to the body’s need for energy or nutrients; it is also a powerful urge for a specific taste, texture, or type of food. Chechlacz et al. (2009) showed stronger activation by food pictures in the insula, orbitofrontal cortex, and basal ganglia of type 2 diabetes patients, which was associated with dietary management. These results are linked to the need for patients with type 2 diabetes to adhere to a lifelong restrictive diet. Considering this study’s results, patients with type 2 diabetes may be more likely to experience stronger food cravings and dietary lapses than healthy adults.

Among social factors, “eating out” predicted “dietary lapse” in patients with type 2 diabetes, while “dinner” and “social situation” predicted “dietary lapse” in healthy adults. “Eating out” was shown to be a high-risk factor in patients with type 2 diabetes. Yamamoto et al. (2000) found that dietary lapses are far more likely to occur on special occasions and in eating-out situations than in everyday life. Inada et al. (2019) also observed using EMA that caloric intake is likely higher in eating-out situations. The current study’s results were similar to those of other studies. Meanwhile, unlike healthy adults, patients with type 2 diabetes did not show an association with social situations. One reason might be that patients with type 2 diabetes in this study had a somewhat long disease duration and could reject others’ recommendations.

Regarding changes in psychological factors before and after eating, “dietary lapse” decreased “vigor,” “fatigue,” and “craving” in type 2 diabetes patients and increased only “vigor” in healthy adults. These results suggest that dietary lapses have different implications for patients with type 2 diabetes compared to healthy adults. For type 2 diabetes patients, the decline in vigor after a dietary lapse may be explained by feelings of self-criticism or guilt associated with a perceived failure to self-regulate. Whittemore et al. (2005) highlighted that diabetes patients frequently struggle with feelings of frustration or failure when they perceive a lapse in self-care behaviors, including diet. Because diabetes management often requires strict dietary control, these patients may consider a dietary lapse as a significant deviation from their health goals, causing their negative emotional response and reduced vigor (Gonzalez et al., 2016). Such emotional response is likely tied to chronic stress and the psychological burden of managing a long-term condition such as diabetes. In contrast, healthy adults experience increased vigor after a dietary lapse, which might be attributed to the absence of strict dietary management and severe health consequences. Unlike diabetes patients, who may associate dietary lapses with self-criticism or health concerns, healthy individuals may interpret these as a permissible method of stress relief, thus improving their emotional state. Moreover, given that social situations are more closely associated with dietary lapses in healthy adults than in type 2 diabetes patients, the improved vigor after a dietary lapse in healthy adults may also be linked to the social enjoyment and pleasure they derive from these situations.

Moreover, these findings suggest that fatigue and cravings may function as establishing operations for dietary lapses in patients with type 2 diabetes. Establishing operations are environmental events or conditions that momentarily alter the effectiveness of a reinforcer and the frequency of behavior that that stimulus has reinforced in the past (Michael, 1982). In this context, elevated levels of fatigue and cravings prior to eating may increase the reinforcing value of food, making it more challenging to adhere to dietary restrictions. The subsequent reduction in these emotions after a lapse may inadvertently reinforce future overeating by creating a temporary sense of relief. In contrast, the absence of such a pattern in healthy adults indicates that establishing operations related to emotions may not exert the same influence on dietary lapses in this population.

Regarding factors affecting daily glucose levels and time in target range, the multivariate model analysis revealed the impact of physical activity, sleep, dietary adherence, and stress on various aspects of glycemic control in patients with type 2 diabetes. The lack of significant associations between time below target (80mg/dl) and the studied factors may be attributed to the characteristics of the study population. The participants in this study were patients with type 2 diabetes without severe complications, potentially representing a population with a relatively low risk of hypoglycemia. Results may differ in patients undergoing insulin therapy or those with longer disease durations.

The latest ADA/EASD consensus report (Buse et al., 2020) emphasized sleep as key to diabetes self-management. Several studies have examined the relation between sleep duration and blood glucose control. In a meta-analysis by Lee et al. (2017), both short sleep duration (<6 h) and long sleep duration (>8 h) were associated with higher HbA1c levels in patients with type 2 diabetes. Similarly, a systematic review by Zhu et al. (2017) found that sleep disturbances, including altered sleep duration, were linked to poorer glycemic control in individuals with type 2 diabetes. The findings of this study indicated a trend of lower average glucose levels on days with longer sleep durations compared with days with shorter sleep durations. This is likely explained by the fact that the participants in this study averaged about 6 h of sleep. While sleep is considered to have three components—quantity, quality, and timing—this study only measured quantity. Hence, future research that evaluates sleep quality and timing may yield new insights. These results show that EMA is a feasible and acceptable method for patients with type 2 diabetes and can provide information on intraindividual variability in psychosocial factors. The real-time assessment tools developed via EMA can be used to inform clinical decision-making and treatment planning. For example, this approach makes it possible to identify specific situations in which individual patients are more likely to engage in unhealthy eating behaviors as well as the psychosocial factors that trigger these behaviors. Spanakis et al. (2016), who performed individual network analyses using EMA data to analyze eating behavior patterns among obese individuals, found that some of these individuals frequently experienced cravings for unhealthy foods, which were strongly associated with actual unhealthy eating. Meanwhile, other individuals were significantly influenced by anxiety, which tends to be exacerbated by unhealthy eating behaviors. These identified eating behavior patterns allows for the development of personalized interventions for each patient. Furthermore, EMA can inform just-in-time adaptive interventions (JITAIs) to address dietary lapses. JITAIs are designed to provide the right type and extent of support at the right time by adapting to an individual’s internal and contextual state (Nahum-Shani et al., 2018). For example, if EMA data indicates that an individual is experiencing high fatigue or craving associated with a greater likelihood of dietary lapse, a JITAI could briefly address that moment via mindfulness or a reminder to engage in self-care activities, among others. Future research should explore how JITAIs could be developed and evaluated to improve dietary management in patients with type 2 diabetes.

Finally, the limitations and future directions of this study must be discussed. First, the participants with type 2 diabetes were limited to outpatients at a local clinic in Japan. Consequently, they had no diabetes complications, and severe cases were not included. These participants’ unique racial/ethnic and diabetic characteristics may limit the generalizability of the results. Second, this study used a single-item rating scale to measure food cravings and minimize respondents’ burden; however, such a method may only partially capture the multidimensional aspects of cravings or trait-based variations. Food cravings are complex experiences involving physiological, cognitive, and emotional components. Furthermore, trait-based aspects of cravings may play a significant role in dietary management; hence, future studies may adopt a more comprehensive measurement approach to elucidate this role in the daily lives of individuals with type 2 diabetes. Third, this study did not consider cognitive factors such as self-efficacy. In a prospective study of Japanese patients with type 2 diabetes, a causal model demonstrated that self-efficacy directly reinforced adherence to diabetes self-care, which was positively correlated with improved glycemic control (Nakahara et al., 2006). Future research may need to assess cognitive factors in these patients. Fourth, this study should have examined the specific content of dietary lapse, which was deemed a subjective indicator; that is, the content likely varied among individuals. Traditional dietary recording approaches have involved paper data, which pose issues such as timeline and content inaccuracies due to batch recording and the complexity of tasks such as calculating intake energy (Inada and Yoshiuchi, 2013). In recent years, however, mobile device dietary recording methods have been developed, highlighting the potential for long-term, low-burden, and accurate dietary records (Fukuo et al., 2009).

## 5 Conclusion

**Figure 1:**
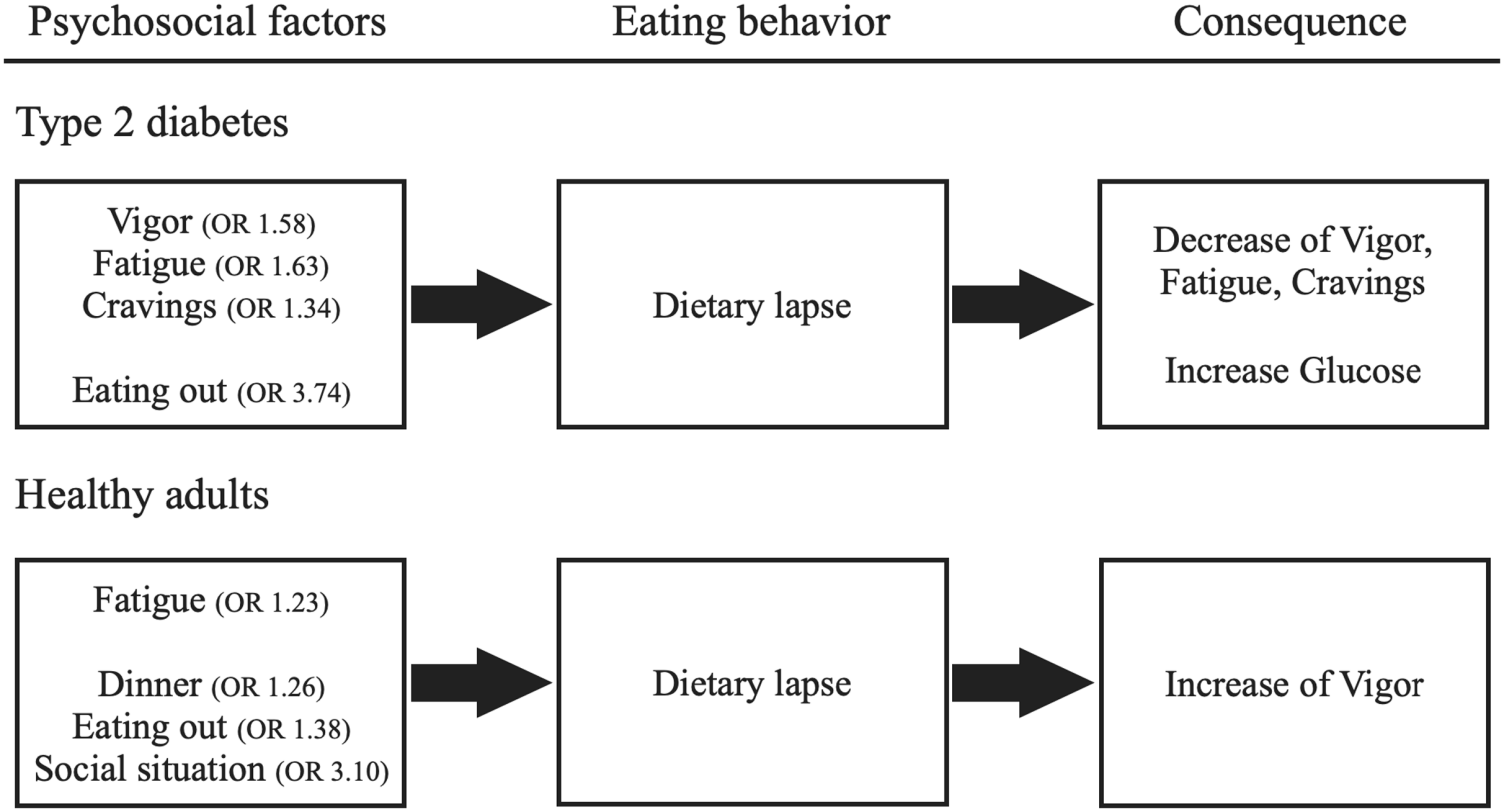
Psychosocial factors that influence dietary lapses and their consequences. The odds ratio (OR) greater than 1 indicated that higher values of the preceding recordings of psychosocial factors were associated with a higher probability of dietary lapse.

This study assessed the relation between psychosocial factors and dietary management in patients with type 2 diabetes using EMA. Its findings showed that dietary lapses in patients with type 2 diabetes were linked to vigor, fatigue, and cravings before eating and the situation of eating out. In contrast, dietary lapses in healthy adults were associated with fatigue before eating and dinner, eating out, and social situations. Regarding changes in psychological factors before and after meals, “dietary lapse” reduced “vigor,” “fatigue,” and “craving” in type 2 diabetes patients. As for fatigue and cravings, these emotions may function as establishing operations, as they are heightened before eating and tend to decrease after dietary lapses. Meanwhile, in healthy adults, dietary lapses increased only vigor, and no identical emotions were both heightened before eating and simultaneously decreased afterward. These findings suggest differences in psychosocial factors that affect dietary management between type 2 diabetes patients and healthy individuals. Figure 1 illustrates these results.

Furthermore, this study emphasized the feasibility and acceptability of EMA as a method for evaluating psychosocial factors and dietary management in daily life in patients with type 2 diabetes. EMA can generate valuable information on intraindividual variability, which can facilitate personalized interventions and JITAIs for improving dietary management.

In conclusion, this study highlighted the importance of considering psychosocial factors in the dietary management of patients with type 2 diabetes. Conducting EMA in clinical settings can provide valuable insights into patients’ daily experiences and inform tailored interventions. Future studies should further investigate the role of psychosocial factors in dietary management and explore how EMA-based interventions can be developed and evaluated to improve diabetes self-management.

## Data Availability

The datasets presented in this article are not readily available because the datasets generated and analyzed are not publicly available due to the regulations of the ethics committee. Requests to access the datasets should be directed to tekuteke@aoni.waseda.jp.
